# Identification of a Novel Papillomavirus Associated with Squamous Cell Carcinoma in a Domestic Cat

**DOI:** 10.3390/v12010124

**Published:** 2020-01-20

**Authors:** Maura Carrai, Kate Van Brussel, Mang Shi, Ci-Xiu Li, Wei-Shan Chang, John S. Munday, Katja Voss, Alicia McLuckie, David Taylor, Andrew Laws, Edward C. Holmes, Vanessa R. Barrs, Julia A. Beatty

**Affiliations:** 1 Sydney School of Veterinary Science, Faculty of Science, University of Sydney, Sydney, NSW 2006, Australia; maura.carrai@sydney.edu.au (M.C.); kate.vanbrussel@sydney.edu.au (K.V.B.); katja.voss@bluewin.ch (K.V.); am5151@cumc.columbia.edu (A.M.); vanessa.barrs@sydney.edu.au (V.R.B.); 2 School of Life and Environmental Sciences and School of Medical Sciences, University of Sydney, Sydney, NSW 2006, Australia; mang.shi@sydney.edu.au (M.S.); cixiu.li@sydney.edu.au (C.-X.L.); wei-shan.chang@sydney.edu.au (W.-S.C.); edward.holmes@sydney.edu.au (E.C.H.); 3 Marie Bashir Institute for Infectious Diseases and Biosecurity, The University of Sydney, Sydney, NSW 2006, Australia; 4 School of Veterinary Science, Massey University, Palmerston North 4410, New Zealand; J.Munday@massey.ac.nz; 5 Vetnostics, 60 Waterloo Rd, North Ryde, NSW 2113, Australia; david.taylor@vetnostics.com.au; 6 Wentworth Falls Animals Hospital, 1/295-297 Great Western Highway, Wentworth Falls, NSW 2782, Australia; fallsvet@commander360.com

**Keywords:** papillomavirus, feline, cat, felid, cancer, squamous cell carcinoma, phylogeny, discovery, pathogen, oncogenic

## Abstract

Papillomaviruses infect the skin and mucosal surfaces of diverse animal hosts with consequences ranging from asymptomatic colonization to highly malignant epithelial cancers. Increasing evidence suggests a role for papillomaviruses in the most common cutaneous malignancy of domestic cats, squamous cell carcinoma (SCC). Using total DNA sequencing we identified a novel feline papillomavirus in a nasal biopsy taken from a cat presenting with both nasal cavity lymphoma and recurrent squamous cell carcinoma affecting the nasal planum. We designate this novel virus as Felis catus papillomavirus 6 (FcaPV6). The complete FcaPV6 7453 bp genome was similar to those of other feline papillomaviruses and phylogenetic analysis revealed that it was most closely related to FcaPV3, although was distinct enough to represent a new viral type. Classification of FcaPV6 in a new genus alongside FcaPVs 3, 4 and 5 is supported. Archived excisional biopsy of the SCC, taken 20 months prior to presentation, was intensely positive on p16 immunostaining. FcaPV6, amplified using virus-specific, but not consensus, PCR, was the only papillomavirus detected in DNA extracted from the SCC. Conversely, renal lymphoma, sampled at necropsy two months after presentation, tested negative on FcaPV6-specific PCR. In sum, using metagenomics we demonstrate the presence of a novel feline papillomavirus in association with cutaneous squamous cell carcinoma.

## 1. Introduction

Papillomaviruses (PVs) are small, nonenveloped, double-stranded DNA viruses that infect the skin and mucosal surfaces of mammals and many other vertebrates in a predominantly host-specific manner. The consequences of papillomavirus infections range from clinically asymptomatic, through benign lesions, to malignant epithelial cancers. The oncogenic potential of papillomaviruses is influenced by virus genotype, host factors such as immune status, and environmental factors, including exposure to sunlight [1].

In domestic cats, five papillomavirus genotypes have been described to date [2]. Felis catus papillomavirus (FcaPV) 1 and FcaPV2 are classified in the *Lambdapapillomavirus* and *Dyothetapapillomavirus* genera, respectively. FcaPV3 and FcaPV4 have been classified in the genus *Taupapillomavirus* although, along with FcaPV5, they have been proposed as members of a new genus, as yet unnamed, on the basis of their L1 open reading frame (ORF) sequence, host species and biological behaviour [3]. 

Feline papillomaviruses are generally thought to be causal in oral papillomas (FcaPV1) [4], viral plaques and Bowenoid in situ carcinomas (BISC) (FcaPV 2, 3 and 5), all of which occur uncommonly. Of greater clinical relevance, a papillomavirus aetiology is suspected for a proportion of cutaneous squamous cell carcinomas (SCC). SCC is the most common feline cutaneous malignancy, comprising 15–48% of all skin tumours in this species [5]. These neoplasms are highly invasive and can cause extensive local tissue destruction. Viral oncogene expression (E6 and E7) and downregulation of tumour suppressor genes *(p53* and *pRb*) link FcaPV2 to a subset of cutaneous SCC, but other papillomaviruses may be contributing [6,7].

Here we report a novel feline papillomavirus—denoted Felis catus papillomavirus 6 (FcaPV6)—that was discovered using metagenomic DNA sequencing of a nasal cavity biopsy from a cat presenting with two nasal cancers, high-grade lymphoma of the nasal cavity and nasal planum SCC.

## 2. Materials and Methods

### 2.1. Clinical Samples

**Figure 1:**
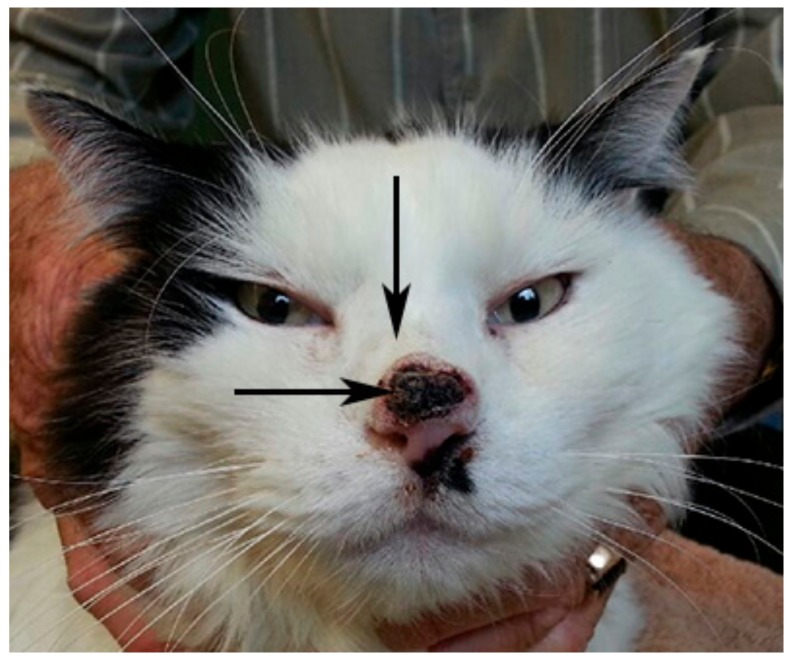
A recurrent invasive squamous cell carcinoma on the nasal planum (horizontal arrow) was adjacent the site of a biopsy (vertical arrow) from which high-grade lymphoma of the nasal cavity was diagnosed.

A 10 year-old male, neutered, domestic, shorthair cat presented with invasive nasal planum SCC that had recurred following excisional biopsy 20 months prior (Figure 1). Concurrent nasal cavity disease was suspected from the history, physical examination and computed tomographic examination of the head. A punch biopsy from the nasal cavity lesion, obtained via a skin incision over the nasal bridge, was divided and stored as formalin-fixed paraffin-embedded (FFPE) tissue and at −80 °C for a virus discovery project with owner consent (approved by the University of Sydney Animal Ethics Committee, 2014/626). The nasal cavity lesion was diagnosed as high-grade B cell lymphoma on histopathology and immunohistochemistry. Retrovirus serology was negative for feline leukaemia virus and positive for feline immunodeficiency virus (FIV), with no prior history of FIV vaccination. Two months later, bilateral renomegaly was detected and euthanasia was requested. Tissues collected at necropsy and stored, as described above, included renal lesions that were subsequently confirmed to be lymphoma. An archived FFPE excisional biopsy, from which the initial diagnosis of nasal planum SCC was made, was retrieved.

### 2.2. DNA Sequencing and Virus Discovery

Total tumour DNA was extracted from the nasal cavity punch biopsy using the DNeasy^®^ Blood and Tissue Kit (Qiagen Pty Ltd., Chadstone, Australia). DNA libraries, constructed using the Nextera XT DNA Library Preparation kit (Illumina, San Diego, CA, USA), were enriched for herpesviruses using customized, hybridization-based target capture kits (myBaits, Arbor Biosciences, Ann Arbor, MI, USA), including a pre-treatment to deplete any remaining streptavidin, according to the manufacturer’s instructions. One set of capture reactions was performed with annealing at 65 °C for 16 h. All PCR amplifications were carried out using KAPA Hi HotStart Mix (Kapa Biosystems, Cape Town, South Africa) with “reamp” primers [8], followed by purification with the GenElute PCR Clean-up kit (Sigma-Aldrich, St Louis, MO, USA). The NextSeq Illumina platform was used to sequence the enriched DNA library. The resulting 150 bp paired-end reads were de novo assembled using Megahit program version 1.1.3 and compared with the nonredundant protein database using Diamond version 0.9.25. This revealed the presence of two distinct contigs of a novel papillomavirus. PCR primers were designed based on recovered reads using Primer3web version 4.1.0 (Table S1) to obtain the full genome sequence. PCR reactions contained 100 ng of template DNA, 200 nM of each primer, 1x Reaction Buffer comprising 1 mM dNTPs and 3 mM MgCl_2_, 1.5 Unit of MyTaq™ HS Red DNA Polymerase (Bioline, Meridian Bioscience, Memphis, TN, USA) in a total volume of 25 µL. Cycling conditions were initial denaturation at 95 °C for 1 min followed by 35 cycles of denaturation at 94 °C for 30 s, primer annealing at 58 °C for 30 s, extension at 72 °C for 30 s and a final extension step at 72 °C for 1 min. The amplicons were sequenced (Macrogen, Seoul, Korea), and Geneious 11.1.5 was used to assemble the contigs to obtain the full genome of this novel papillomavirus.

### 2.3. Phylogenetic Analysis

To determine the evolutionary relationships of the novel papillomavirus identified here, we inferred a phylogenetic tree on the basis of the concatenated alignment of four protein sequences (E1, E2, L2 and L1). Accordingly, the relevant amino acid sequences of 66 papillomaviruses, representative of the full genetic diversity of these viruses in mammals (as well as six nonmammalian papillomaviruses used as outgroups to root the phylogeny), were aligned using the E-INS-I algorithm in the MAFFT v7 package [9]. Ambiguously aligned regions were removed using GBlocks [10]. This resulted in a final sequence alignment of 850 amino acid residues. A phylogenetic tree was then estimated using the maximum likelihood method in PhyML 3.0 [11], employing the LG+Γ model of amino acid substitution and a subtree pruning and regrafting (SPR) branch-swapping algorithm. Topological robustness was assessing using SH-like branch supports.

### 2.4. Investigation of Disease Association

The novel papillomavirus sequences could have originated from the SCC located on the nasal planum immediately adjacent the punch biopsy site (Figure 1), the nasal cavity lymphoma, which was the primary target of the biopsy, or other adjacent tissues. A potential association between the novel papillomavirus and the SCC or the lymphoma was therefore investigated as follows:

#### 2.4.1. DNA Extraction from FFPE SCC Biopsy

DNA was extracted from scrolls of FFPE tissue cut at 8 microns using standard techniques to avoid contamination using the DNeasy^®^ Blood and Tissue Kit.

#### 2.4.2. Papillomavirus-Specific PCR

A conventional polymerase chain reaction (cPCR) assay specific for the novel papillomavirus was performed on DNA extracted from the archived FFPE SCC biopsy, and from frozen renal lymphoma obtained at necropsy using primer sets Pap_F4 and Pap_F7 (Table S1). DNA extracted from the punch biopsy was used as the positive control and no-template (molecular-grade water) as the negative control in all PCR assays. Products were separated on 1.0% agarose gel containing SYBRTM Safe DNA Gel Stain (Invitrogen, Carlsbad, CA, USA) in Tris–Borate–EDTA (TBE) 1× buffer and were visualised under UV light (Bio-Rad Gel Doc XR System, Bio-Rad Laboratories Pty Ltd., Gladesville, Australia). The identity of bands migrating at the expected size was confirmed using Sanger sequencing. 

#### 2.4.3. p16 Immunostaining 

Sections of 5 micron thickness were cut from the archived SCC biopsy onto charged slides (Superfrost Plus, Thermoscientific, Waltham, MA, USA). Immunostaining using anti-p16^CDKN2A^ protein (p16) antibodies (p16INK4 clone G175-405, BD Pharmingen, Franklin Lakes, NJ, USA) was performed as previously described [12].

#### 2.4.4. Papillomavirus Consensus PCR

To detect other papillomaviruses in the nasal planum SCC, DNA extracted from the FFPE biopsy was examined by PCR using consensus primers FAP59/64, MY09/11 and CP4/5, which were designed to amplify DNA from a wide range of human cutaneous and mucosal papillomavirus [4]. The JMPF/R primers, which specifically amplify FcaPV2 DNA, were also used [13]. DNA extracted from a BISC that contained FcaPV2 DNA was the positive control for the FAP59/64 and JMPF/R primers, while DNA extracted from a BISC that contained FcaPV3 was the positive control for the MY09/11 and CP4/5 primers.

## 3. Results and Discussion

### 3.1. Identification of Felis Catus Papillomavirus 6

**Figure 2:**
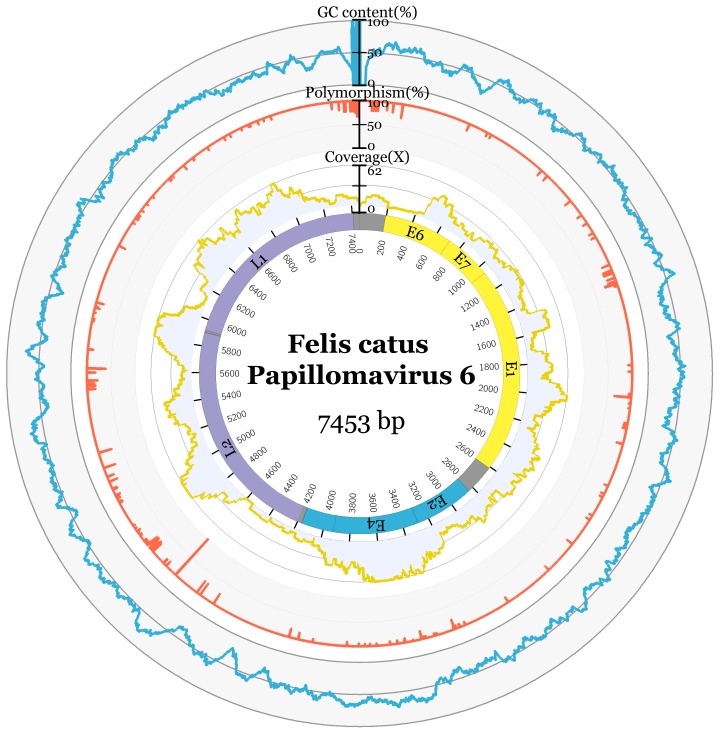
Felis catus Papillomavirus 6 (FcaPV6) genome configuration using metadata. The predicted ORFs are represented by the coloured inner segments. GC content is displayed in blue, percentage nucleotide polymorphism in orange and read coverage in yellow.

**Figure 3:**
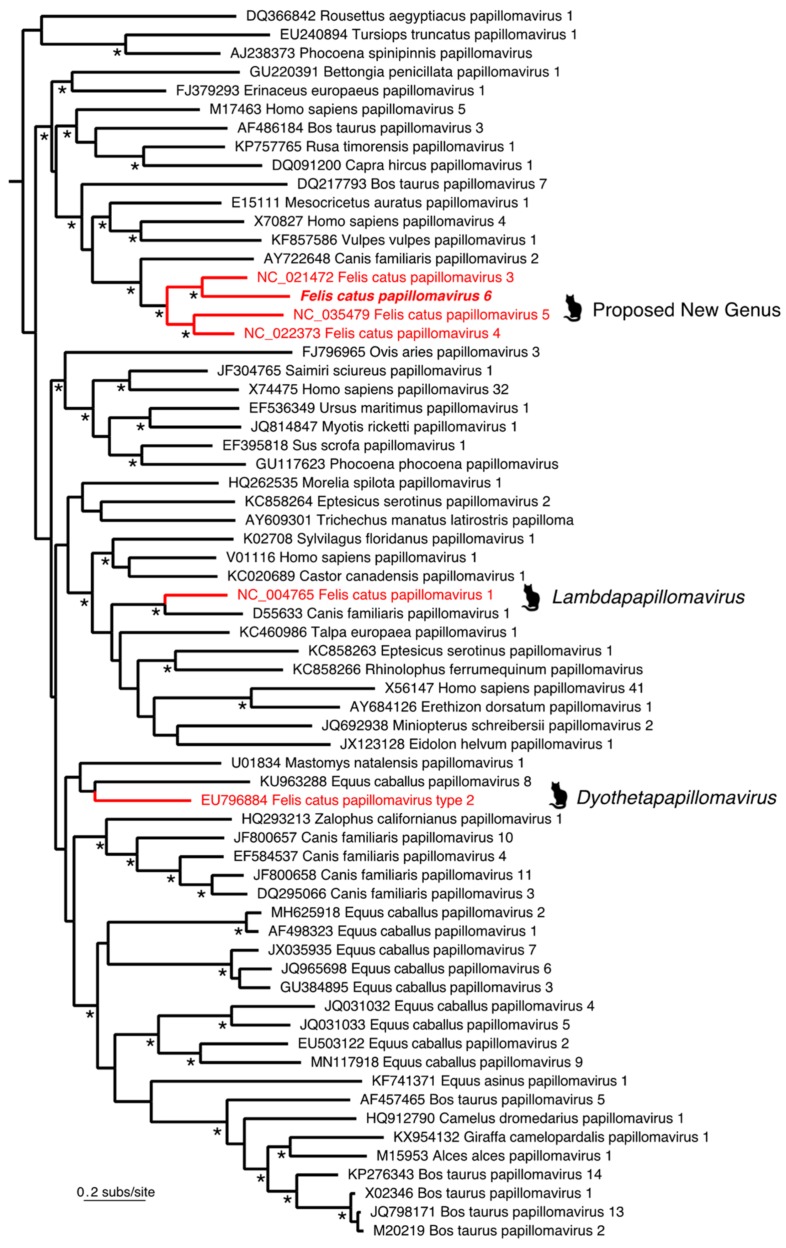
Evolutionary relationships of FcaPV6 to other mammalian papillomaviruses, including those previously identified in cats (*Felis catus*). Those papillomaviruses previously identified in cats are marked with animal symbols and in red, along with their respective genera. FcaPV6 is proposed to fall as part of a proposed new genus. All virus names contain their associated GenBank accession numbers. The tree is rooted using six nonmammalian papillomaviruses that have been removed to improve clarity. All horizontal branch lengths are scaled according to the number of amino acid substitutions per site, and the * denotes nodes with SH-like branch support values >0.95.

Two distinctive papillomavirus contigs of lengths 2847 bp and 4184 bp were identified from metagenomic sequencing of a DNA library originally enriched for herpesvirus discovery. The sequence occupying the gaps between the two fragments was obtained by PCR to form a circular genome of 7453 bp in length and confirmed with PCR. Reads were mapped back to the full FcaPV6 genome and showed an abundance level of 153.7 RPM (reads per million) (Figure 2). Blast analyses based on the entire genome suggest the virus was clearly related to those of Felis catus papillomavirus 3, 4 and 5 observed previously, with the novel papillomavirus identified here most closely related to FcaPV3. Papillomaviruses are classified based on the sequence of the highly conserved ORF L1, with PVs within the same genera sharing over 60% nucleotide similarity and PVs of the same type having over 90% similarity [14,15]. The ORF L1 nucleotide sequence of the novel papillomavirus was most similar to FcaPV3. However, as the two sequences were only 66.1% similar (Table 1), this is consistent with the identification of a new papillomavirus type, and a classification of FcaPV6 is proposed. The FcaPV6 L1 ORF was 60.5% similar to FcaPV4 and 59.6% similar to FcaPV5. Recently, FcaPV3, 4 and 5 were proposed to be members of a novel distinct papillomavirus genus that infects the skin of domestic cats [3]. Due to the similarity of the FcaPV6 ORF L1 to these three feline papillomavirus types, it is proposed that FcaPV6 joins FcaPV3, 4 and 5 within this proposed new genus. Importantly, this pattern of relationship was strongly confirmed by the phylogenetic analysis (Figure 3), with FcaPV6 and FcaPV3 grouping together, and with strong support for a cat-specific cluster of papillomaviruses (i.e., FcaPV3, FcaPV4, FcaPV5 and FcaPV6). The complete genome sequence of FcaPV6 has been submitted to GenBank and assigned accession number MN857145.

### 3.2. Genome Characteristics

The full genome of FcaPV6 has a 48.9% GC content. Seven ORFs were predicted to encode for two late proteins, L1 and L2, and five early proteins, E1, E2, E4, E6 and E7 (Figure 2). The predicted ORF features are displayed in Table 2. The E2 binding site, identified by the sequence ACC-N(5-7)-GGT was predicted at twelve positions in the genome, in addition to three NF1 CGGAA and Sp1 binding sites GGCGGG and four polyadenylation sites AATAAA (Table S2; Figure S1). Zinc-binding domains CXXC-X29-CXXC were predicted in E6 (84, 157 aa), separated by 36aa, and E7 (75 aa) (Table S3; Figure S1). The ATP-binding site for the ATP-dependent helicase GPPNTGKS and cyclin RXL motif KRRLF in E1 and nuclear localisation signal in L2 RKRRR and L1 KRKR were predicted in FcaPV6 (Table S3; Figure S1).

### 3.3. Association of FcaPV6 with Squamous Cell Carcinoma

**Figure 4:**
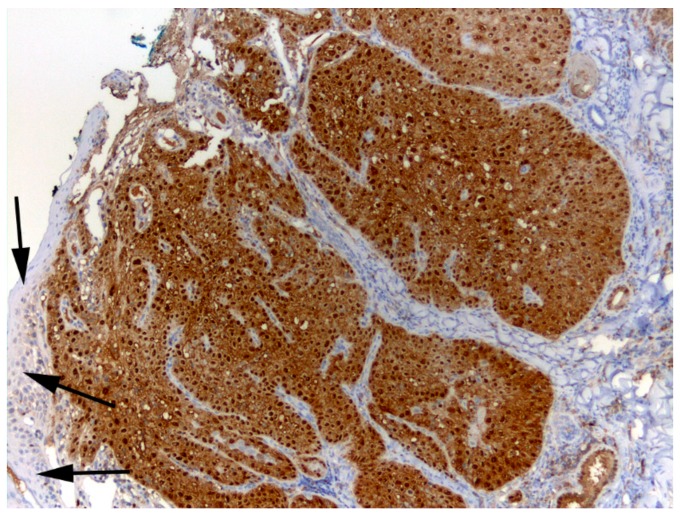
Photomicrograph of a nasal planum squamous cell carcinoma that contained FcaPV6 DNA. This biopsy was obtained 20 months prior to the SCC recurrence shown in Figure 1. Intense nuclear and cytoplasmic immunostaining using anti-p16^CDKN2A^ protein (p16) antibodies is visible diffusely within the neoplastic cells. There is minimal immunostaining within adjacent non-neoplastic epidermis (arrows). Hematoxylin counterstain.

The excisional biopsy of the nasal planum SCC showed diffuse intense p16 immunostaining (Figure 4). Overexpression of p16 is a surrogate marker for a papillomaviral aetiology in SCC in humans and cats so this result supports a papillomavirus aetiology for the SCC [12,16]. DNA extracted from the same SCC biopsy tested positive for FcaPV6 on two virus-specific cPCRs, generating products of 558 bp and 191 bp, thereby confirming that this virus was associated with the nasal planum SCC at least 20 months prior to presentation. However, no additional PV types were amplified from the SCC using either consensus primers or primers specific for FcaPV2. Therefore, FcaPV6 was the only papillomavirus identified in association with the SCC. Consensus primers have been used to discover FcaPV 1 to 5 [3] but failed to detect FcaPV6 in DNA extracted from the SCC. This could have been because of sequence diversity noted at the primer binding sites. If the commonly used consensus primers do have low sensitivity for FcaPV6, this would explain why this papillomavirus type has not previously been detected in any feline cutaneous preneoplastic or neoplastic lesion. Alternatively, if FcaPV6 was present at low copy number, it is conceivable virus-specific primers would be more likely to generate a PCR product than consensus primers. Regardless, the failure of the consensus primers to detect FcaPV6 demonstrates the utility of unbiased metagenomic sequencing to discover divergent organisms. While the p16 immunostaining and detection of only FcaPV6 within the lesion supports a potential causal association between this papillomavirus and the SCC, most papillomavirus infections are asymptomatic and FcaPV6 may, like many other papillomaviruses of humans and animals, constitute part of the normal virome [17,18]. Investigation of a larger number of cutaneous lesions as well as normal skin from cats is required to better understand the relationship, if any, of FcaPV6 to cutaneous neoplasia in cats. 

Evidence supporting an association between papillomavirus infection and a diagnosis of lymphoma is limited [19]. Nonetheless, given that the novel virus sequences were identified in a biopsy targeting a high-grade lymphoma, it was decided to test lymphoma tissue arising at a site distant to the nasal planum in the same patient for the presence of FcaPV6. No papillomaviral DNA was amplified from DNA extracted from a renal lymphoma lesion obtained at necropsy using FcaPV6-specific primers Pap-_4 and Pap_7.

## 4. Conclusions

This study identifies a novel papillomavirus genotype, designated FcaPV6, in the presence of squamous cell carcinoma of the nasal planum in a domestic cat. The biological niche and disease associations of FcaPV6 clearly merit additional investigation. The application of unbiased virus discovery methodologies to lesions suspected to have a papillomavirus aetiology may advance pathogen discovery.

## Figures and Tables

**Table 1:** Percentage pairwise nucleotide sequence identities of feline papillomaviruses in the L1 ORF.

Virus	FcaPV2	FcaPV1	FcaPV3	FcaPV6	FcaPV5	FcaPV4
FcaPV2		55.7	56.8	55.2	56.1	55.1
FcaPV1	55.7		56.4	54.2	56.5	57.4
FcaPV3	56.8	56.4		66.1	62.8	63.8
FcaPV6	55.2	54.2	66.1		59.6	60.5
FcaPV5	56.1	56.5	62.8	59.6		64.4
FcaPV4	55.1	57.4	63.8	60.5	64.4	

**Table 2:** ORF characteristics of Felis catus Papillomavirus 6 (FcaPV6).

ORF	Start	End	Length (nt)	Length (aa)	GC%
L1	5919	7409	1491	497	46.5
L2	4261	5901	1641	547	54.4
E1	1059	2858	1800	600	44.8
E2	2836	4167	1332	444	51.2
E4	3287	3922	636	292	57.4
E6	195	785	591	197	48.2
E7	372	707	1078	124	50.0

## Supplementary Materials

The following are available online at https://www.mdpi.com/1999-4915/12/1/124/s1, Figure S1: Felis catus Papillomavirus 6 (FcaPV6) genome configuration and nucleotide (nt) and amino acid (aa) feature location. Table S1: Oligonucleotides used to amplify the complete genome of Felis catus papillomavirus 6 (FcaPV6), Table S2: Predicted nucleotide features of Felis catus papillomavirus 6 (FcaPV6), Table S3: Predicted amino acid features of Felis catus papillomavirus 6 (FcaPV6).

## References

[1] McBride A.A. (2017). Oncogenic human papillomaviruses. Philos. Trans. R. Soc. B Biol. Sci..

[2] Munday J.S., Sharp C.R., Beatty J.A. (2019). Novel viruses: Update on the significance of papillomavirus infections in cats. J. Feline Med. Surg..

[3] Munday J.S., Dittmer K.E., Thomson N.A., Hills S.F., Laurie R.E. (2017). Genomic characterisation of Felis catus papillomavirus type 5 with proposed classification within a new papillomavirus genus. Vet. Microbiol..

[4] Munday J.S., French A.F. (2015). Felis catus papillomavirus types 1 and 4 are rarely present in neoplastic and inflammatory oral lesions of cats. Res. Vet. Sci..

[5] Gross T.L., Ihrke P., Walder E.J., Affolter V.K. (2005). Epidermal Tumors. Skin Diseases of the Dog and Cat.

[6] Hoggard N., Munday J.S., Luff J. (2018). Localization of Felis catus Papillomavirus Type 2 E6 and E7 RNA in Feline Cutaneous Squamous Cell Carcinoma. Vet. Pathol..

[7] Altamura G., Corteggio A., Pacini L., Conte A., Pierantoni G.M., Tommasino M., Accardi R., Borzacchiello G. (2016). Transforming properties of Felis catus papillomavirus type 2 E6 and E7 putative oncogenes in vitro and their transcriptional activity in feline squamous cell carcinoma in vivo. Virology.

[8] Meyer M., Kircher M. (2010). Illumina sequencing library preparation for highly multiplexed target capture and sequencing. Cold Spring Harb. Protoc..

[9] Katoh K., Standley D.M. (2013). MAFFT Multiple Sequence Alignment Software Version 7: Improvements in Performance and Usability. Mol. Biol. Evol..

[10] Talavera G., Castresana J. (2007). Improvement of Phylogenies after Removing Divergent and Ambiguously Aligned Blocks from Protein Sequence Alignments. Syst. Biol..

[11] Guindon S., Gascuel O. (2003). A Simple, Fast, and Accurate Algorithm to Estimate Large Phylogenies by Maximum Likelihood. Syst. Biol..

[12] Munday J.S., Knight C.G., French A.F. (2011). Evaluation of feline oral squamous cell carcinomas for p16CDKN2A protein immunoreactivity and the presence of papillomaviral DNA. Res. Vet. Sci..

[13] Munday J.S., Kiupel M., French A.F., Howe L. (2008). Amplification of papillomaviral DNA sequences from a high proportion of feline cutaneous in situ and invasive squamous cell carcinomas using a nested polymerase chain reaction. Vet. Dermatol..

[14] Bernard H.-U., Burk R.D., Chen Z., van Doorslaer K., Hausen H.Z., de Villiers E.-M. (2010). Classification of papillomaviruses (PVs) based on 189 PV types and proposal of taxonomic amendments. Virology.

[15] de Villiers E.M., Fauquet C., Broker T.R., Bernard H.U., zur Hausen H. (2004). Classification of papillomaviruses. Virology.

[16] Parry D., Bates S., Mann D.J., Peters G. (1995). Lack of cyclin D-Cdk complexes in Rb-negative cells correlates with high levels of p16INK4/MTS1 tumour suppressor gene product. EMBO J..

[17] Antonsson A., Erfurt C., Hazard K., Holmgren V., Simon M., Kataoka A., Hossain S., Hakangard C., Hansson B.G. (2003). Prevalence and type spectrum of human papillomaviruses in healthy skin samples collected in three continents. J. Gen. Virol..

[18] Munday J.S., Witham A.I. (2010). Frequent detection of papillomavirus DNA in clinically normal skin of cats infected and noninfected with feline immunodeficiency virus. Vet. Dermatol..

[19] Intaraphet S., Farkas D.K., Johannesdottir Schmidt S.A., Cronin-Fenton D., Søgaard M. (2017). Human papillomavirus infection and lymphoma incidence using cervical conization as a surrogate marker: A Danish nationwide cohort study. Hematol. Oncol..

