# Supplementary material for: Identification of a Novel Papillomavirus Associated with Squamous Cell Carcinoma in a Domestic Cat
Source: Viruses. 2020 Jan 20;12(1):124. doi: 10.3390/v12010124 (PMC7019393; doi:10.3390/v12010124)
Supplement: Supplementary file 1 [file viruses-12-00124-s001.zip › viruses-689474-Figure S1.pdf]

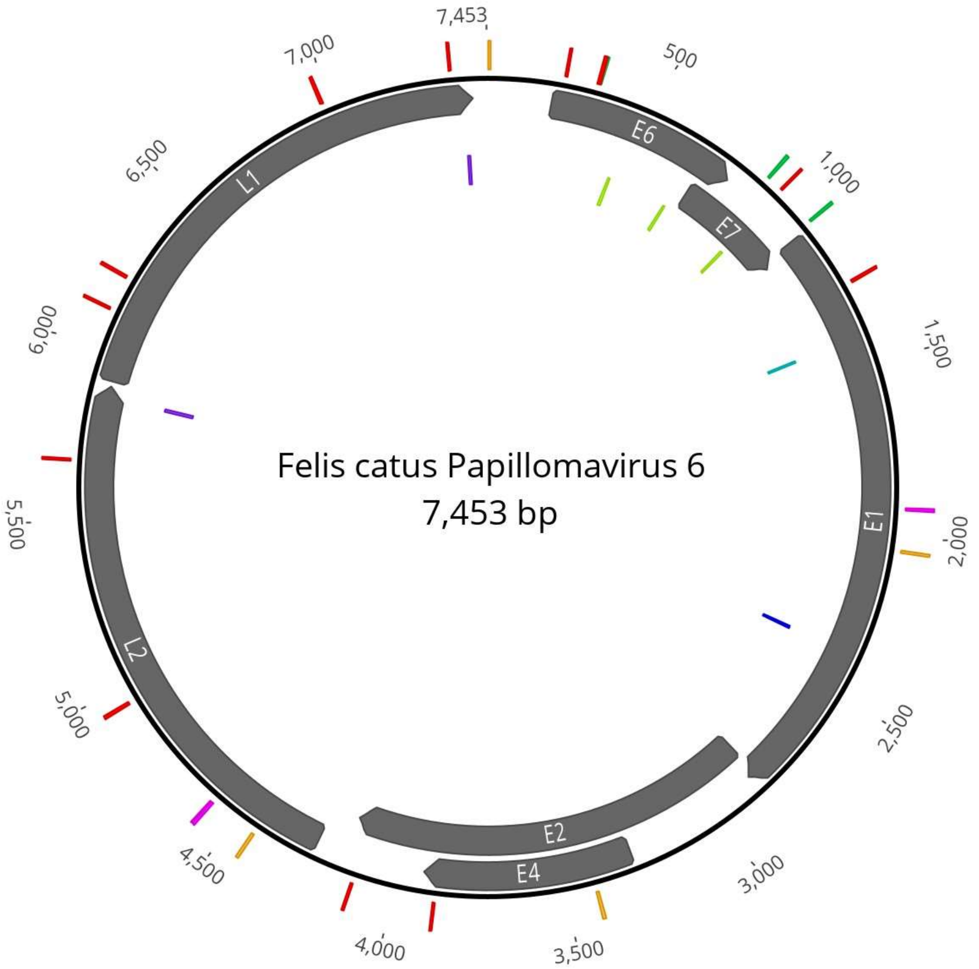

Predicted feature (nt)	Sequence
E2 binding sites	ACC-N4-GGT
	ACC-N5-GGT
	ACC-N6-GGT
	ACC-N7-GGT
Polyadenylation sites	AATAAA
Sp1 binding sites	GGCGGG
NF1 binding sites	CGGAA
AP1 binding site	TGANTCA

*All motif sites displayed inside ORFs

Predicted feature (aa)	Translation
ATP-dependent helicase motif	GPPNTGKS
Cyclin interaction RXL motif	KRRLF
Metal-binding motifs	CXXC-X29-CXXC
Metal-binding motifs	CXXC-X29-CXXC
Nuclear localisation signal	RKRRR
Nuclear localisation signal	KRKR
Retinoblastoma binding domain	LXCXE

*All binding sites displayed outside ORFs

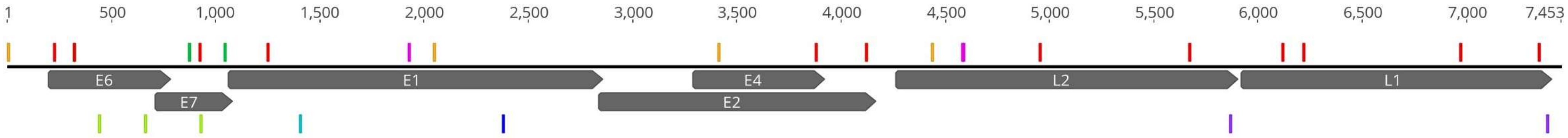

Figure S1 Felis catus Papillomavirus 6 (FcaPV6) genome configuration and nucleotide (nt) and amino acid (aa) feature location.
