# Supplementary material for: Identification of a Novel Papillomavirus Associated with Squamous Cell Carcinoma in a Domestic Cat
Source: Viruses. 2020 Jan 20;12(1):124. doi: 10.3390/v12010124 (PMC7019393; doi:10.3390/v12010124)
Supplement: Supplementary file 1 [file viruses-12-00124-s001.zip › viruses-689474-Supplementary Tables.docx]

Table S1. Oligonucleotides used to amplify the complete genome of Felis catus papillomavirus 6 (FcaPV6)

| **Oligonucleotides** | **Sequence** | **Start -5'** | **End -3'** | **Tm (°C)** | **Size (bp)** |
| --- | --- | --- | --- | --- | --- |
| Pap_F4 | TCGTCCAAGTCAACCACGAG | 7359 | 7378 | 60 | 558 |
| Pap_R4 | TAGCCCCCACAAAAGCAACA | 463 | 444 | 60 | 558 |
| Pap_F5 | TTGGGTCAGAGGTCACAAGG | 7326 | 7345 | 59 | 555 |
| Pap_R5 | TGAACAGGAATCCTCAGCCG | 427 | 408 | 60 | 555 |
| Pap_F6 | TAAAAGACGCAAGCGGGACT | 4359 | 4378 | 60 | 303 |
| Pap_R6 | ATAGTGTCCACCGGGCCTAT | 4661 | 4642 | 60 | 303 |
| Pap_F7 | AGATTGGTAGCAGCCTCACT | 4478 | 4497 | 58 | 191 |
| Pap_R7 | ACTGCCTATAGTGTCCACCG | 4668 | 4649 | 58 | 191 |

**Table S2.** Predicted nucleotide features of Felis catus papillomavirus 6 (FcaPV6).

| **Predicted feature (nt)** | **Sequence** | **ORF** | **Occurrence** | **nt position** |
| --- | --- | --- | --- | --- |
| E2 binding sites | ACC-N4-GGT | All | 3 | 1251, 4122, 7350 |
|  | ACC-N5-GGT |  | 1 | 3881 |
|  | ACC-N6-GGT |  | 5 | 226, 321, 923, 5672, 6219 |
|  | ACC-N7-GGT |  | 3 | 4953, 6121, 6973 |
| Polyadenylation sites | AATAAA | NC, E1, E2, L2 | 4 | 4, 2049, 3415, 4438 |
| Sp1 binding sites | GGCGGG | E1, L2 | 3 | 1928, 4582, 4586 |
| NF1 binding sites | CGGAA | E6, E7 | 3 | 323, 876, 1043 |
| AP1 binding site | TGANTCA | - | - | - |

**Table S3.** Predicted amino acid features of Felis catus papillomavirus 6 (FcaPV6).

| **Predicted feature (aa)** | **Translation** | **ORF** | **Occurrence** | **aa position** |
| --- | --- | --- | --- | --- |
| ATP-dependent helicase motif | GPPNTGKS | E1 | 1 | 441 |
| Cyclin interaction RXL motif | KRRLF | E1 | 1 | 117 |
| Metal-binding motifs | CXXC-X29-CXXC | E6 | 2 | 84, 157 |
| Metal-binding motifs | CXXC-X29-CXXC | E7 | 1 | 75 |
| Nuclear localisation signal | RKRRR | L2 | 1 | 537 |
| Nuclear localisation signal | KRKR | L1 | 1 | 491 |
| Retinoblastoma binding domain | LXCXE | - | - | - |
